# Participant Engagement With a Digital Behavioral Health App for Chronic Pain: Descriptive Secondary Analysis of a Feasibility Randomized Controlled Trial

**DOI:** 10.2196/88122

**Published:** 2026-04-09

**Authors:** Susan M Zbikowski, Jo Masterson, Yohali Burrola-Mendez, Chialing Hsu, Kris Pui Kwan Ma, Ying Zhang, Deanna Waters, Kari A Stephens

**Affiliations:** 1 2Morrow, Inc, 12020 113th Ave NE, Ste 295, Kirkland, WA, 98034, United States, 1 833-344-8425; 2 inZights Consulting, LLC, Seattle, WA, United States; 3 Department of Family Medicine, University of Washington, Seattle, WA, United States

**Keywords:** chronic pain, self-management, digital health, behavioral health, acceptance and commitment therapy

## Abstract

**Background:**

Chronic pain is a widespread condition that impairs quality of life and is often managed primarily with medications. National guidelines now recommend nonpharmacologic, mind-and-body, and behavioral approaches as first-line or complementary treatments. However, access to these evidence-based options remains limited. Digital health technologies offer a scalable way to deliver integrative, self-care interventions that empower patients to live well with pain.

**Objective:**

This study examined engagement with and perceived usefulness of a patient- and health care professional–informed mobile app designed to deliver behavioral and educational content to support pain self-management.

**Methods:**

Adult primary care patients with chronic pain were enrolled in a 12-week feasibility trial. The app included lessons addressing the physical, emotional, and social aspects of pain; tracking and personalized insights; self-screenings; and optional in-app coaching. Participants completed baseline and 3-month surveys assessing usability and satisfaction. Engagement was evaluated through app analytics and milestone completion.

**Results:**

Of 49 patients assigned to the app, 40 (81.6%) activated it. Participants used the app for an average of 27.3 (SD 25.2) unique days and completed an average of 25.5 (SD 22.5) core lessons. Engagement highlights included 42.5% (17/40) completion of the valued living module, 25.0% (10/40) completion of all lessons, and 50.0% (20/40) use of daily check-ins. Usability ratings were high, with 86.7% (26/30) reporting that the app helped them better understand or manage their pain and 90.0% (27/30) recommending it to others.

**Conclusions:**

Adults with chronic pain engaged with the program and reported high satisfaction with this evidence-informed digital mind-and-body intervention. Findings from this feasibility study suggest the potential for digital tools to support access to nonpharmacologic, integrative pain self-care and complement traditional clinical approaches.

## Introduction

Chronic pain is one of the most common reasons why adults seek medical care [1]. It affects over 50 million adults in the United States—more than 20% of the population—with approximately 8.5% experiencing pain that limits daily functioning, including participation in work and social activities [2]. The burden is not equally distributed; rates are higher among individuals with lower income and educational levels and those with public health insurance [3]. Chronic pain is associated with long-term opioid use, emotional distress, and disability [4,5] and is estimated to cost the US economy between US $560 and US $635 billion annually in direct health care expenditures and lost productivity [6].

In response to the opioid epidemic, national guidelines recommend behavioral interventions as first-line or complementary treatments for chronic pain [7,8]. Evidence supports cognitive behavioral therapy, acceptance and commitment therapy, and mindfulness-based interventions [9-11], yet these treatments are underused in routine care. Primary care providers, often the first point of contact for patients [12], face barriers to implementing these strategies, including limited training in behavioral approaches for pain; time constraints; and system-level barriers such as insufficient insurance coverage, access challenges, and limited availability of trained professionals [12-14]. Because most behavioral pain treatments are delivered in specialty settings, scalability and accessibility are limited [13-15]. The National Academy of Medicine has called for the development of innovative strategies to reduce these barriers and support more equitable and effective pain care [12].

Digital health technologies, including mobile apps, can extend the reach of evidence-based behavioral interventions by delivering support asynchronously outside traditional clinical settings and being integrated into broader care models. They align with needs to diversify service delivery and equip medical professionals, including those in primary care, with accessible tools for pain management [13,16-18]. However, reviews show that many consumer apps lack credible content or health care professional input, have limited functionality, and fail to engage users [19,20]. This highlights the need for engaging, evidence-based, patient- and health care professional–informed apps that address the full biopsychosocial experience of pain and can be used in clinical practice.

We conducted a feasibility study of a self-directed mobile app designed to deliver evidence-based educational content and behavioral strategies to adults with chronic pain. This study evaluated participant engagement, including patterns of use, frequency of use, and feature use. Insights into user interaction with digital behavioral tools are essential for refining intervention design, informing clinical integration, and guiding future research and scale-up.

## Methods

### Study Design

We analyzed engagement data collected during a feasibility randomized control trial of a mobile behavioral health app designed to help patients treated in primary care for chronic pain management. The feasibility trial was a 2-arm randomized controlled trial designed to assess the feasibility of trial implementation (recruitment, randomization, treatment, and retention) and explore preliminary impacts on pain, pain interference, and mental health. This paper presents a descriptive analysis of user experience and app use data among participants in the intervention arm, who had access to the mobile behavioral health app during the study period. Participants were randomized using a stratified approach based on health care system and age to ensure balanced allocation across health systems and approximate the national age distribution of adults living with chronic pain.

### Ethical Considerations

This study was conducted as part of a National Institutes of Health Small Business Innovation Research–funded collaboration, with 2Morrow Inc serving as the grant recipient and developer of the intervention and the University of Washington serving as the academic research partner.

All study procedures, including recruitment, consent, data collection, and follow-up, were reviewed and approved by the University of Washington Institutional Review Board (STUDY00016340) and were conducted in accordance with the approved study protocols and applicable federal regulations. All patients provided informed consent prior to participation. Patient privacy and confidentiality were protected through multiple safeguards. Data were stored on secure, access-controlled systems, and all analytic datasets were deidentified prior to analysis. No directly identifying information was included in analytic files. App use data were captured automatically through the intervention platform and linked to study data using unique participant identifiers. Data handling procedures complied with applicable privacy and confidentiality requirements. Patients assigned to the Salty program were provided with 2Morrow’s privacy policy at the time of program activation, which outlines how user data are collected, used, and protected.

Patients received a US $50 electronic gift card for each completed survey (up to US $200 total).

### Recruitment and Eligibility

Primary care patients with chronic pain were identified through electronic health records at 4 health systems (60 clinics) in Washington and Idaho, sent an email invitation to participate in the study, and directed to complete a screening survey to determine eligibility. Eligible participants had at least one documented chronic pain condition identified through electronic health records. Types of pain represented in the study population included back pain, neck pain, noninflammatory joint pain, abdominal or bowel pain, fibromyalgia, and headache disorders. Patients were selected to participate if they were actively receiving care for their chronic pain (≥2 primary care visits in the previous year), currently endorsed struggling with chronic pain for 3 months or longer, endorsed having pain interference in their lives (≥55 on the Patient-Reported Outcomes Measurement Information System Pain Interference scale [21,22]), could use an app in English, and had a smartphone compatible with the behavioral health app.

**Figure 1.**
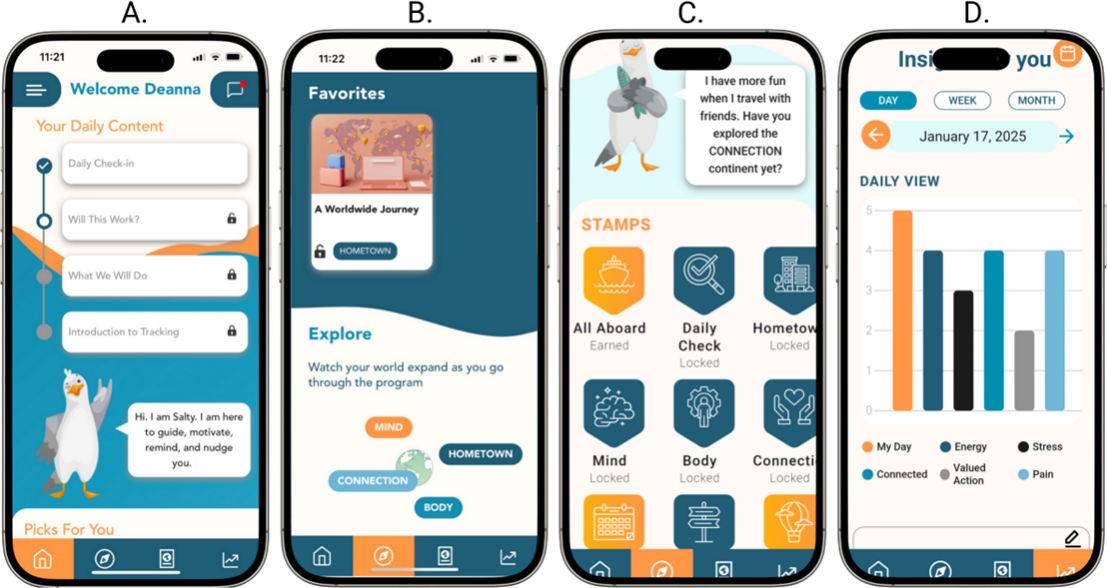
Screenshots of the pain app: (A) daily task recommendations, (B) the section where patients can explore other core lesson categories, (C) the passport stamp page for viewing completed milestones, and (D) the tool that provides insights from the daily check-in data.

Patients randomized to the intervention group received access to the Salty for Chronic Pain app, a novel digital behavioral health intervention developed by 2Morrow Inc (Figure 1). To be included in this study and analysis, patients had to successfully activate the app, which included downloading it and completing their in-app personal profile.

Patients were invited to participate via email and directed to an online interest survey. The survey provided 3 options: proceed to eligibility screening, request a call from a study coordinator for additional information, or opt out of the study. Patients who completed the eligibility screening were informed of their eligibility status at the end of the survey. Those who were eligible were directed to an electronic consent form, which allowed them to confirm participation, request further discussion with a study coordinator, or decline participation. Patients who confirmed participation provided informed consent electronically prior to enrollment.

### Program Design and Features

Salty for Chronic Pain (pain app) is a 12-week self-directed program designed to educate and support patients managing the emotional, social, and behavioral aspects of living with chronic pain with the goal of reducing pain interference and improving function and quality of life. The program was created with input from patients, health care professionals, and pain behavioral health specialists during phase 1 of the Small Business Innovation Research trial; health care professionals indicated that they wanted additional options for their patients with pain that were evidence based but did not require much of their time [23].

The program used the metaphor of guiding patients on a personal journey to explore the physical, emotional, and social aspects of pain to learn how to live life despite having pain. The program included a total of 58 core lessons divided into 4 sections and additional bonus content. The first core section, known as “Hometown,” included 22 lessons related to the concept of valued living and living with pain based on principles from acceptance and commitment therapy. The other sections covered the physical (16 lessons), emotional (13 lessons), and social (7 lessons) aspects of pain.

The program included tools for gaining personal insights into pain and daily functioning. The “Daily Check-in” allowed patients to rate their overall day (“awesome” to “awful”), as well as energy, activity, stress, social connection, valued activities, and pain (all on 5-point scales), with results displayed graphically across daily, weekly, or monthly views. A journal feature was provided for recording additional reflections, and patients could optionally connect a Fitbit (provided at no cost) to track activity, sleep, and heart rate. Self-screenings were available in the “Self-Discovery” section, including the Depression, Anxiety, and Stress Scale-21 (DASS-21 [24]); Valued Living Questionnaire (VLQ; [25]); Drug Use Disorders Identification Test (DUDIT [26]); and Alcohol Use Disorders Identification Test (AUDIT [27]). Although the program was intended to be self-guided, participants could message with a coach on the app about lessons, personal discoveries, and progress. These features and others on the app are described in Textbox 1.

Textbox 1. **Summary of core features and functionality on the app.**

**Profile**

- Description: pain-related questions that patients complete to set up their account
- Purpose: benchmark data and personalization

**Core lessons**

- Description: 58 short lessons related to 4 domains (valued living—acceptance and commitment therapy concept, physical aspects of pain, emotional aspects of pain, and social aspects of pain)
- Purpose: pain information, education, and coping skills

**Bonus lessons**

- Description: additional content that patients could access
- Purpose: additional education, tips, and patient stories

**Daily check-in**

- Description: a brief daily questionnaire for patients to rate various aspects of living with pain
- Purpose: a personal pain tracking tool

**Journal**

- Description: patients could record their pain experience in their own words
- Purpose: a flexible and personal pain tracking and insight tool

**Personalized messaging**

- Description: brief in-app messages
- Purpose: provided to direct, motivate, or reinforce concepts

**Sensor integration**

- Description: participants could link a Fitbit device to the app to track additional insights
- Purpose: objective tracking of sleep, activity, and heart rate

**Self-screening questionnaires**

- Description: 4 standard questionnaires for patients to explore alcohol and drug use, mental health, and valued living
- Purpose: to explore and better understand their substance use, mental health, and what is most important to them

**Insight dashboard**

- Description: a visualization tool to explore pain insights
- Purpose: ability to view the interconnection among self-reported pain, energy, activity, stress, social connection, and other metrics

**Milestone achievement**

- Description: participants could receive up to 12 stamps on their passport page
- Purpose: summarize and reward progress on the app

**Monthly check-in**

- Description: a brief monthly questionnaire
- Purpose: in-app survey to measure pain, interference, mental health, quality of life, and satisfaction each month after a patient enrolls in and uses the program

**Message a coach**

- Description: asynchronous chat tool with a coach
- Purpose: meant to provide additional support and encouragement for patients who wanted to connect with a coach; patients could share insights and achievements as well

**Navigation and pacing features**

- Patients were instructed to complete a certain number of lessons along with a daily check-in each day that they visited the app
- The app required completion of all Hometown lessons before moving on to another section
- Patients needed to complete each core section before selecting another one to complete
- Patients could complete the self-screening tools only once per month
- Save a favorite: a feature on the app to save content that participants found helpful

### How the Intervention Worked

Each time patients opened the app, they were prompted to complete a “Daily Check-in” and up to 3 scheduled lessons and could view optional bonus content. They were encouraged to engage with the app 2 to 3 times per week over 12 weeks, with lesson access paced to discourage rapid completion and promote sustained use. All participants began with valued living content in the “Hometown” section before advancing to the “Social,” “Physical,” or “Emotional” modules. Completion of all lessons within a category was required before moving on as content was designed to build sequentially. Participants could bookmark favorite lessons and retained access to app content and features for 100 days.

### App Milestone Completion

Patients earned up to 12 milestone stamps (Table 1) as a gamified feature to promote engagement and track progress. Stamps were displayed on the “Passport” tab and awarded for completing the profile, core content (values, body, mind, connection, and bonus), at least 10 “Daily Check-ins,” and a monthly check-in, as well as for connecting a Fitbit, messaging a coach, completing self-screenings, and visiting all recommended sections.

**Table 1.** Passport stamps for the 12 milestones completed on the app (N=40).

Milestone	Stamp name	Participants receiving the stamp, n (%)
Completion of profile	All Aboard	40 (100.0)
Completion of daily check-in 10 times	Daily Check	20 (50.0)
User visited the “Self-Discovery,” “Passport” (stamp location), “Explore” navigator tool, and “Insights” pages	Traveler	19 (47.5)
One self-screening (alcohol or substance use, depression and anxiety screening, or valued living questionnaire) completed	Discover	17 (42.5)
Hometown core content completed (22 lessons)	Hometown	17 (42.5)
Mind core content completed (13 lessons)	Mind	11 (27.5)
Body core content completed (16 lessons)	Body	13 (32.5)
Connection core content completed (7 lessons)	Connection	11 (27.5)
15 bonus lessons completed	Extras	14 (35.0)
First monthly check-in completed	Monthly Check	14 (35.0)
One engagement with a coach	Coach	13 (32.5)
Synchronizing the Fitbit	Sync Up	5 (12.5)

### Data Collection

Demographics (age, gender, race and ethnicity, educational level, and employment status) and pain history (type and duration) were collected at screening or baseline. Use metrics were captured from the app data system. App metrics included app use milestones achieved (eg, earned passport stamps) and use metrics, including unique days of use, duration (days between first and last use), core lessons completed, feature use (tracking and self-screenings), and in-app actions.

User experience was measured at 3 months via the mHealth App Usability Questionnaire (MAUQ [28]) and a custom perceived usefulness survey developed by 2Morrow, Inc. Acceptability and usefulness were derived from the MAUQ Ease of Use and Satisfaction Domain (Q1-8). The custom survey asked patients to rate the helpfulness of the app overall and of specific features, value, and the likelihood that they would recommend the app to others.

Recruitment, screening, and survey administration were managed by the University of Washington. Patients had 2 weeks to complete each survey.

### Analyses

Descriptive statistics (frequencies, means, ranges, and percentages) were calculated for demographics and engagement, describing the types of app content, features, and tools accessed.

## Results

### Study Population

Of the 49 patients randomized to the pain app intervention arm, 40 (81.6%) met the inclusion criteria for this study (ie, successfully activated the program by downloading the app and completing their profile). Participants reported living with chronic pain for 3 or more years; were aged 47.3 (SD 17.3) years on average (the most common age group was 30-44 years); and identified as women for the most part followed by men, with a smaller proportion (3/40, 7.5%) identifying as nonbinary. Nearly half (19/40, 47.5%) of the participants identified as White individuals, with the remainder (21/40, 52.5%) identifying as Asian, Pacific Islander, or Hawaiian; Black; multiracial; or other. Educational attainment ranged from high school to postgraduate education, with most participants (32/39, 82.1%) reporting at least some college education. At baseline, a substantial proportion of the participants reported a history of mental health conditions, reflecting the clinical complexity of the study population. Employment status varied, including part-time work, full-time employment, and disability status. Counts and percentages are reported in Table 2.

**Table 2.** Participant characteristics (N=40).

	Participants, n (%)
Gender	
Women	25 (62.5)
Men	12 (30.0)
Nonbinary	3 (7.5)
Race or ethnicity	
Asian American	5 (12.5)
Black or African American	5 (12.5)
Native Hawaiian or other Pacific Islander	2 (5.0)
Two or more races	6 (15.0)
White	19 (47.5)
Other	3 (7.5)
Age group (years)	
<30	8 (20.0)
30‐44	12 (30.0)
45-64	12 (30.0)
≥65	8 (20.0)
Educational level (n=39)	
Lower than high school	3 (7.7)
High school graduate or GED^a^	4 (10.3)
Some college but did not complete degree	5 (12.8)
2- or 4-year degree	10 (25.6)
More than 4-year college degree	17 (43.6)
Employment status (n=38)	
Employed full time	15 (39.5)
Employed part time	5 (13.2)
Contract or temporary	1 (2.6)
Voluntarily unemployed	3 (7.9)
Involuntarily unemployed	1 (2.6)
Unable to work or disabled	8 (21.1)
Retired	5 (13.2)
Pain duration	
3 months to 1 year	3 (7.5)
1 year to 3 years	5 (12.5)
≥3 years	32 (80.0)

a GED: General Educational Development.

### Outcome Measures

#### App Use

##### Passport Stamps Earned

All participants earned at least one passport stamp, with a mean of 4.9 (SD 3.8; range 1‐12) stamps per user, indicating broad engagement with the app. The most frequently earned stamps reflected onboarding and routine use, including completing the profile (40/40, 100.0%), completing at least 10 daily check-ins (20/40, 50.0%), and exploring multiple app sections (19/40, 47.5%). A total of 42.5% (17/40) completed at least one core content module, with all of these participants completing the valued living (“Hometown”) module; in this subgroup, 58.8% (10/17) completed all 4 content modules, and 82.4% (14/17) accessed bonus content. Overall, 25.0% (10/40) of the participants earned 9 or more stamps, suggesting a subset engaged deeply with multiple features, whereas others demonstrated more selective use.

##### Lesson Completion and Days of Use

**Figure 2.**
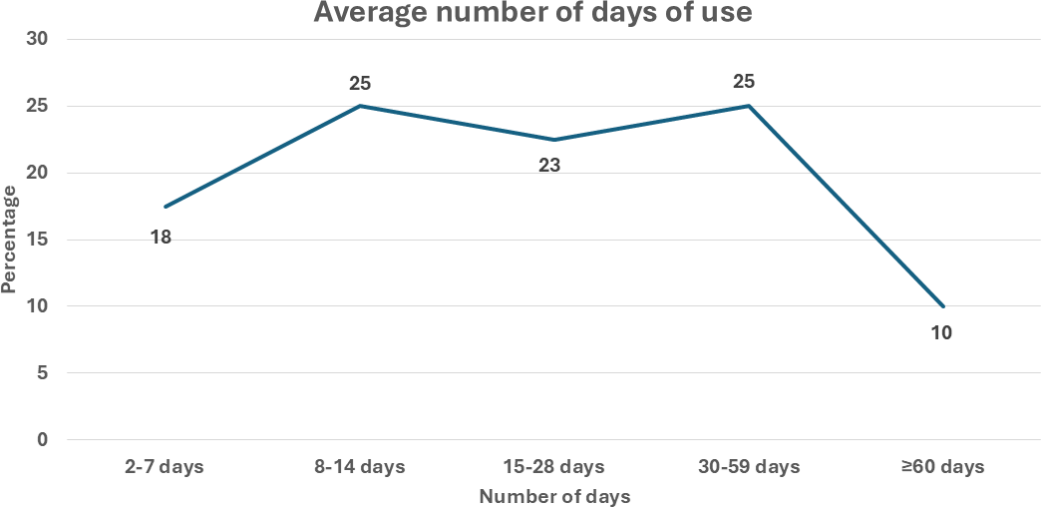
Unique days of app use (N=40).

Participants completed a mean of 25.5 (SD 22.5, range 2‐58) core lessons. A total of 42.5% (17/40) completed the valued living module, and 25.0% (10/40) completed all 58 core lessons across the 4 content areas. Participants engaged with the app on an average of 27.3 (SD 25.2, range 2‐118) unique days over the 12-week period; 57.5% (23/40) used the app on 15 or more days, and 75.0% (30/40) interacted with the app across a span of 30 to 100 calendar days (Figure 2).

##### Additional Engagement Indicators

Use of tracking and optional features varied. In total, 82.5% (33/40) completed at least one milestone related to pain insight activities (daily check-ins, self-screenings, or monthly check-ins), with over 40% (17/40, 42.5%) completing at least one mental health or substance use self-screening. Completion rates for each self-screening ranged from 22% to 30%: DUDIT (9/40, 22.5%), VLQ (10/40, 25%); AUDIT (10/40, 25.0%), and DASS-21 (12/40, 30.0%). A smaller proportion engaged with coaching features (13/40, 32.5%) or synchronized a Fitbit device (5/40, 12.5%). Across all users, mean in-app activity totaled 2123 (SD 2579.8, range 48‐10,332) actions, reflecting substantial variability in depth of interaction.

### User Survey Results

#### Overview

The response rate to the follow-up survey at 3 months was 85.0% (34/40) for the MAUQ ease of use and satisfaction domain questions (Table 3). Due to an error in the survey build, the custom perceived usefulness survey response rate was somewhat lower (30/40, 75.0%), and missing responses were mostly due to random missingness (Table 4).

**Table 3.** Frequency distribution of participant responses to the mHealth App Usability Questionnaire ease of use and satisfaction domain items (N=34).

Item	Strongly disagree, n (%)	Disagree, n (%)	Somewhat disagree, n (%)	Neither agree nor disagree, n (%)	Somewhat agree, n (%)	Agree, n (%)	Strongly agree, n (%)	Somewhat agreed, agreed, or strongly agreed, n (%)
The app was easy to use	0 (0.0)	0 (0.0)	2 (5.9)	5 (14.7)	2 (5.9)	15 (44.1)	11 (32.4)	28 (82.4)
It was easy for me to learn to use the app	0 (0.0)	0 (0.0)	2 (5.9)	3 (8.8)	2 (5.9)	14 (41.2)	13 (38.2)	29 (85.3)
I like the interface of the app^a^	0 (0.0)	1 (3.0)	3 (9.1)	6 (18.2)	2 (6.1)	11 (33.3)	10 (30.3)	23 (69.7)
The information in the app was well organized, so I could easily find the information I needed	0 (0.0)	0 (0.0)	3 (8.8)	6 (17.6)	10 (29.4)	6 (17.6)	9 (26.5)	25 (73.5)
I feel comfortable using this app in social settings	2 (5.9)	2 (5.9)	1 (2.9)	6 (17.6)	7 (20.6)	6 (17.6)	10 (29.4)	23 (67.6)
The amount of time involved in using this app has been fitting for me	1 (2.9)	2 (5.9)	3 (8.8)	4 (11.8)	4 (11.8)	10 (29.4)	10 (29.4)	24 (70.6)
I would use this app again^a^	1 (3.0)	0 (0.0)	2 (6.1)	5 (15.2)	4 (12.1)	10 (30.3)	11 (33.3)	25 (75.8)
Overall, I am satisfied with this app	1 (2.9)	0 (0.0)	1 (2.9)	8 (23.5)	4 (11.8)	9 (26.5)	11 (32.4)	24 (70.6)

a Results are reported based on 33 valid responses.

**Table 4.** Frequency distribution of participant responses to the pain app perceived usefulness survey (N=30).

Item	Not at all, n (%)	Slightly, n (%)	Moderately, n (%)	Very, n (%)	Extremely, n (%)	Slightly, moderately, very, or extremely, n (%)
The Salty Program helped me with my pain, for example, to better understand or manage my pain	4 (13.3)	7 (23.3)	11 (36.7)	6 (20.0)	2 (6.7)	26 (86.7)
Was using the Salty program worth your time?	4 (13.3)	6 (20.0)	8 (26.7)	9 (30.0)	3 (10.0)	26 (86.7)
Please rate how helpful the following features in the Salty Program were to you:						
The lessons and content in the program	3 (10.0)	4 (13.3)	9 (30.0)	11 (36.7)	3 (10.0)	27 (90.0)
Having the program divided into different topics ranging from physical, emotional, social aspects of pain	4 (13.3)	3 (10.0)	3 (10.0)	14 (46.7)	6 (20.0)	26 (86.7)
Having content related to how pain may affect you physically	4 (13.3)	4 (13.3)	4 (13.3)	15 (50.0)	3 (10.0)	26 (86.7)
Having content related to how pain may affect you emotionally	4 (13.3)	3 (10.0)	3 (10.0)	13 (43.3)	7 (23.3)	26 (86.7)
Having content related to how pain may affect you socially	4 (13.3)	4 (13.3)	4 (13.3)	13 (43.3)	5 (16.7)	26 (86.7)
How much you got to do each day	5 (16.7)	6 (20.0)	8 (26.7)	10 (33.3)	1 (3.3)	25 (83.3)
Having to complete an entire section of lessons before moving to other topics or themes	7 (23.3)	6 (20.0)	6 (20.0)	9 (30.0)	2 (6.7)	23 (76.7)
Daily check-ins to track how things are going for you	3 (10.0)	6 (20.0)	5 (16.7)	9 (30.0)	7 (23.3)	27 (90.0)
Viewing insights from daily tracking	5 (16.7)	4 (13.3)	7 (23.3)	10 (33.3)	4 (13.3)	25 (83.3)
The option to connect with a coach	8 (26.7)	4 (13.3)	9 (30.0)	6 (20.0)	3 (10.0)	22 (73.3)
Earning passport stamps	9 (30.0)	8 (26.7)	7 (23.3)	3 (10.0)	3 (10.0)	21 (70.0)
Opportunity to receive a Fitbit	9 (30.0)	3 (10.0)	9 (30.0)	4 (13.3)	5 (16.7)	21 (70.0)
Seeing Fitbit data in the app	10 (33.3)	3 (10.0)	9 (30.0)	2 (6.7)	6 (20.0)	20 (66.7)
Length of the program (100 d)	3 (10.0)	4 (13.3)	10 (33.3)	12 (40.0)	1 (3.3)	27 (90.0)
Self-discovery surveys in the app to learn more about your mood, drinking, etc	5 (16.7)	6 (20.0)	6 (20.0)	5 (16.7)	8 (26.7)	25 (83.3)
Videos that you watched	4 (13.3)	9 (30.0)	8 (26.7)	5 (16.7)	4 (13.3)	26 (86.7)

#### MAUQ Ease of Use and Satisfaction Domains

Overall usability and satisfaction ratings were favorable (Table 3). Most participants reported that the app was easy to learn (29/34, 85.3%) and use (28/34, 82.4%). More than 70% (25/34, 73.5%) agreed that the app was well organized and the time commitment was appropriate, were satisfied overall, and would use it again.

#### Custom Perceived Usefulness Survey

Core features—including daily check-ins; structured lessons; and content addressing the physical, emotional, and social aspects of pain—were rated as helpful by 76.7% (23/30) to 90.0% (27/30) of the participants. While features such as receiving passport stamps, Fitbit integration, and coach messaging received somewhat lower ratings, more than two-thirds (20/30, 66.7%) still found them useful. Overall, 90% (27/30) of respondents reported that they would recommend the app to others. Additionally, 23.3% (7/30) rated the daily check-ins and emotional content as extremely helpful, with comparable ratings across other content areas (Table 4).

## Discussion

### Principal Findings

This study evaluated the Salty for Chronic Pain app, a behavioral digital health program designed to support adults living with chronic pain. Unlike most studies on digital health programs for pain that report only frequency or duration of use, our evaluation incorporated milestone completion, lesson progression, self-tracking, and perceptions of usefulness, offering a more comprehensive view of engagement and potential value as a complement to clinical care. Patients demonstrated sustained engagement for nearly a month, advanced through structured lessons, and reported high usability and satisfaction, suggesting that the program appeared feasible and was generally well received. These findings add to a growing body of research showing that digital platforms can deliver behavioral pain self-management strategies in ways that are usable, acceptable, and aligned with evidence-based approaches [10,16,23,29].

Although direct comparisons to other studies are limited due to differences in design, content, and metrics, our findings are broadly consistent with those of prior digital pain intervention research [30,31]. Activation rates were high, suggesting that patients could begin using the app with minimal support, which may help reduce burden on clinical staff. Most participants met recommended frequency targets, indicating that the app design supported regular use without intensive reminders. These results are comparable to those of Thomson et al [32], who reported that 77% of participants achieved minimum engagement during a 6-week intervention.

On average, patients completed more than 25 core lessons, and engagement extended beyond the introductory module, with a subset completing all lessons and others using bonus content. This level of engagement is noteworthy given that lessons were sequenced, requiring completion of one section before progressing, and the valued living module (“Hometown”) was mandatory before others could be accessed. In addition, no automated reminders were delivered due to a technical issue. Hence, engagement occurred without automated reminders, suggesting that participants were self-motivated. It is possible that greater use would have been achieved with automated reminders or more flexible sequencing. At the same time, these findings raise important questions about how much content exposure is necessary to achieve meaningful improvements in pain, acceptance, and functioning. Some patients may benefit from a targeted subset of lessons, whereas others may require more extensive exposure. Clinical outcomes will be reported separately. Future refinements, such as adding flexible sequencing or automated prompts, may increase completion rates and help identify the “dose” of content most strongly associated with clinical benefit or perceived value.

These findings highlight the potential of digital behavioral health tools to support access to evidence-based pain self-management strategies. Participants engaged with structured lessons and interactive features that promoted reflection, self-monitoring, and awareness of valued living—processes associated with acceptance, coping, and resilience in chronic pain populations [33,34]. The results suggest that such strategies can be delivered in digital form with high usability and satisfaction; however, engagement metrics reflect exposure to content rather than therapeutic dose or clinical effect.

Several limitations should be considered when interpreting these findings. The relatively small sample size reflects the feasibility nature of the trial and limits generalizability to all adults with chronic pain. The eligibility criteria and study context further shape interpretation: participants were required to have smartphone access, English-language proficiency, and clinically significant pain interference, and most reported long-standing pain. Accordingly, the sample may overrepresent individuals who were digitally ready or motivated to engage with a self-directed program. The engagement patterns observed in this study may not generalize to all adults with chronic pain, particularly those with limited digital access or differing readiness to engage in behavioral interventions.

Taken together, these findings suggest that this type of digital behavioral health program may be particularly well suited for individuals who are motivated to engage in self-directed care and interested in nonpharmacologic or complementary approaches to chronic pain management. The observed engagement without automated reminders further suggests potential fit for users who prefer flexible, low-burden support delivered outside traditional clinical settings.

Engagement and satisfaction ratings may have also been influenced by study participation, incentives associated with survey completion, novelty effects, or app design constraints such as fixed lesson sequencing.

Despite these limitations, this study provides early evidence that an evidence-informed, user-centered mobile program can be feasibly delivered and meaningfully engaged with by adults receiving care for chronic pain. These findings can inform intervention refinement and support the design of larger studies to evaluate engagement patterns; predictors of sustained use; and effects on pain interference, functioning, and quality of life [17,18].

### Conclusions

Patients successfully activated the program without support, most met recommended use targets, and many engaged deeply with the available content and insight tools. These findings are promising for the use of self-directed evidence-based digital behavioral health apps in clinical pain management. While patients rated the features favorably, additional research is needed to determine how much exposure is necessary to achieve clinical benefit. Future studies should evaluate the impact of these tools on pain interference and functioning, identify engagement patterns linked to improved outcomes, and test refinements such as flexible sequencing or automated reminders. Addressing these questions can help digital behavioral health programs expand access; supplement usual care; and support more equitable, scalable approaches to chronic pain management.

## Abbreviations

AUDIT: Alcohol Use Disorders Identification Test
DASS-21: Depression, Anxiety, and Stress Scale-21
DUDIT: Drug Use Disorders Identification Test
MAUQ: mHealth App Usability Questionnaire
VLQ: Valued Living Questionnaire
